# Hiding in Plain Sight: HIV-1 Membraneless Organelles as Nuclear Hubs—Host Hijacking, Replication, Immune Evasion, and Drug-Access Implications

**DOI:** 10.3390/pathogens15070766

**Published:** 2026-07-21

**Authors:** Francesco Broccolo, Alessandro Sannino, Mauro Pollini, Federica Paladini, Thierry Mourer, Francesca Di Nunzio

**Affiliations:** 1 Department of Experimental Medicine (DiMeS), University of Salento, 73100 Lecce, Italy; alessandro.sannino@unisalento.it (A.S.); mauro.pollini@unisalento.it (M.P.); federica.paladini@unisalento.it (F.P.); 2 UOSD Microbiology and Virology, P.O. Vito Fazzi, ASL Lecce, 73100 Lecce, Italy; 3 Advanced Molecular Virology Unit, Department of Virology, Institut Pasteur, Université Paris Cité, 75015 Paris, France; thierry.mourer@pasteur.fr (T.M.); francesca.di-nunzio@pasteur.fr (F.D.N.)

**Keywords:** HIV-1, biomolecular condensates, membraneless organelles, CPSF6, nuclear speckles, phase separation, host hijacking, cGAS–STING, restriction factors, innate immune evasion, viral replication, viral reservoir, antiretroviral drug penetration, condensate partitioning, capsid inhibitors, lenacapavir

## Abstract

Theories on the early steps of the HIV-1 life cycle have been radically revised over the past five years. The long-held assumption that the capsid fully disassembles in the cytoplasm has given way to a more nuanced view: Cytoplasmic disassembly does occur and, in several myeloid systems, is increasingly linked to cytosolic cDNA sensing and to abortive infection. However, a substantial fraction of intact or nearly intact capsid cores instead traverse the nuclear pore complex (NPC) and, upon interacting with the host factor CPSF6, induce liquid–liquid phase separation. This leads to the formation of biomolecular condensates, termed HIV-1 membraneless organelles (HIV-1-MLOs), which subsequently merge with nuclear speckles (NSs). In this Perspective we read these condensates along five interlocking axes. First, the virus drives the host phase separation of cleavage and polyadenylation specificity factor 6 (CPSF6), which quickly fuses with another MLO: the NS composed of the speckle scaffold factors, SON and SRRM2. Second, the resulting condensate behaves as a catalytic site that concentrates the reverse-transcription machinery and thereby promotes integration of the viral DNA into speckle-associated chromatin (SPADs). Third, the same compartment is the final layer of a stratified programme of innate immune evasion, shielding nascent double-stranded DNA from cGAS–STING after cytoplasmic restriction factors and sensors have been outmanoeuvred. Fourth, although demonstrated only in vitro, stable HIV-1-MLOs can maintain the viral RNA genome in the presence of a reverse-transcription inhibitor. Upon removal of the inhibitor, reverse transcription resumes, mirroring, to some extent, the situation in individuals undergoing interruption of antiretroviral therapy and suggesting that these structures may act as a pre-integration reservoir. Fifth, and still largely unexplored, the sanctuary has a pharmacological dimension: anatomical lymphoid compartments, and possibly the condensate itself through selective small-molecule partitioning, may limit antiretroviral drug access. We situate HIV-1-MLOs within the convergent condensate strategies of SARS-CoV-2 and other viruses, and we discuss the clinical, diagnostic, therapeutic, and vaccine implications, including capsid inhibitors as “block-and-expose” tools.

## 1. Introduction

Since the isolation of the retrovirus responsible for AIDS [1], and despite more than four decades of research and roughly 40 million people living with HIV worldwide [2], the molecular choreography of the earliest steps of infection has only recently come into focus. For most of that period a single model dominated the textbooks: the conical capsid, built from about 1500 copies of the capsid (CA) protein and enclosing two copies of the single-stranded RNA genome, was thought to disassemble in the cytoplasm shortly after fusion; reverse transcription was assumed to be completed there; and only the pre-integration complex (PIC), carrying fully double-stranded viral DNA, was believed to enter the nucleus for integration [3]. This view was internally coherent but, as it turned out, largely wrong about where and when the decisive events occur.

The first crack appeared when CA was identified as a dominant determinant of lentiviral nuclear import in non-dividing cells [4]. A succession of studies then showed that reverse transcription is not an exclusively cytoplasmic event: nuclear import of the core precedes the completion of reverse transcription, and transcription-competent double-stranded DNA can be generated entirely within the nucleus [5,6,7]. Cryo-electron tomography demonstrated that cone-shaped capsids traverse intact nuclear pore complexes (NPCs) [8,9], and that uncoating, far from being an early cytoplasmic event, occurs in the nucleus, close to sites of integration, through the release of viral cDNA from capsid-like structures [10]. The corollary is that the host nucleus, not the cytosol, is the stage on which the early viral life cycle is decided. This nuclear emphasis does not deny cytoplasmic uncoating: both subcellular-fractionation and single-particle imaging studies document capsid disassembly in the cytosol, and in some myeloid contexts, cytoplasmic DNA sensors can detect the resulting viral cDNA. Current evidence indicates, however, that cytoplasmic uncoating is frequently coupled with abortive, non-productive infection, whereas the cores that establish productive infection tend to preserve capsid integrity until they reach the nuclear compartment.

In parallel, cell biology supplied an organising principle for these nuclear events. Membraneless organelles (MLOs), or biomolecular condensates, have been recognised as pervasive regulators of cellular and viral processes [11,12,13]. Incoming HIV-1 genomes were found to accumulate in nuclear niches enriched in cleavage and polyadenylation specificity factor 6 (CPSF6), where reverse transcription is clustered [14,15]; these CPSF6 puncta behave as bona fide condensates, dispersed by 1,6-hexanediol and hypertonic stress, and they are important for productive infection [16,17]. Collectively these structures are now referred to as HIV-1 membraneless organelles (HIV-1-MLOs).

Three recent reports have substantially advanced this picture; because they originate largely from the same collaborative group, we present them as convergent evidence that will benefit from independent confirmation. A hypothesis-driven synthesis frames HIV-1-MLOs as the crossroads of nuclear reverse transcription and innate immune evasion [18]; an in vivo study demonstrates that these condensates form in infected humanised mice, license nuclear reverse transcription, and safeguard the viral genome against cGAS [19]; and a mechanistic dissection identifies the disordered domains of CPSF6 and of the nuclear-speckle scaffold SRRM2 that govern condensate biogenesis and fusion [20]. A fourth, complementary study introduces an imaging platform that tracks individual HIV-1 DNA molecules from cell culture to the tissues of humanised mice, resolving transcribing from silent genomes across lymphoid organs [21]. In this Perspective we integrate these advances with the condensate literature of the past three years, organising the discussion around five interlocking themes (host hijacking, replication, immune evasion, latency, and antiretroviral drug access) before drawing cross-viral parallels and weighing the clinical, diagnostic, therapeutic, and vaccine implications.

## 2. A New Model of Early HIV-1 Nuclear Events: Condensates as Functional Organelles

**Figure 1:**
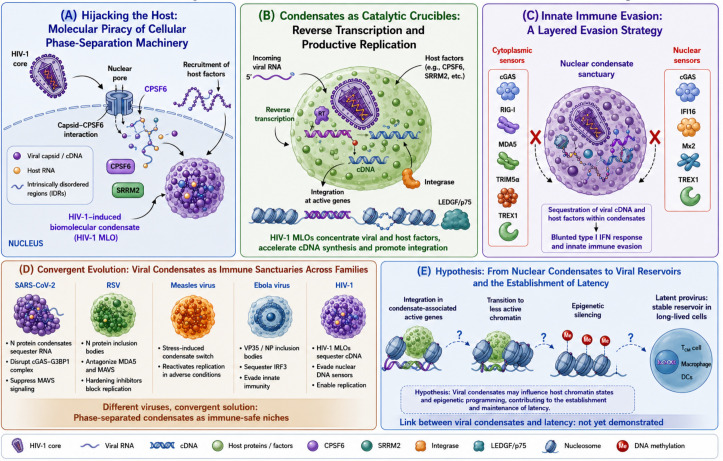
Nuclear events: condensates as functional organelles. Following entry into the nucleus, the HIV-1 capsid exploits the host phase separation machinery to generate CPSF6-dependent biomolecular condensates (HIV-1 membraneless organelles, HIV-1-MLOs), which function as specialised nuclear compartments coordinating multiple early steps of the viral life cycle. (**A**) Hijacking the host. The viral capsid traverses the FG-nucleoporin phase of the nuclear pore complex and recruits CPSF6, SRRM2, LEDGF/p75, and other host phase separation proteins to nucleate HIV-1-induced condensates. (**B**) Condensates as catalytic crucibles. These condensates concentrate viral cores, carrying reverse transcriptase, integrase, and viral genomes, with host cofactors, thereby promoting efficient completion of reverse transcription, formation of the pre-integration complex, and preferential integration into transcriptionally active, speckle-associated chromatin. (**C**) Innate immune evasion. HIV-1-MLOs function as nuclear immune sanctuaries by sequestering newly synthesised viral double-stranded DNA from innate immune sensors, including the cGAS–STING pathway, while complementing restriction-evasion mechanisms involving TREX1, SAMHD1, MX2, TRIM5, and IFI16. (**D**) Convergent viral evolution. Similar phase-separated condensates have independently evolved in unrelated viral families, including SARS-CoV-2, respiratory syncytial virus (RSV), measles virus, and Ebola virus, where they promote genome replication while suppressing innate immune recognition, illustrating convergent evolution toward condensate-mediated immune-protected replication niches. (**E**) Hypothesis: Establishment of viral reservoirs and latency. Following productive reverse transcription and integration, HIV-1-MLOs could potentially contribute to the formation of transcriptionally silent viral reservoirs through chromatin remodelling and epigenetic silencing, providing the initial nuclear sanctuary that seeds long-lived latent infection in memory CD4^+^ T cells, macrophages, and dendritic cells. Collectively, HIV-1-induced condensates emerge as multifunctional organelles that integrate host hijacking, productive replication, immune evasion, and potentially latency establishment, redefining the earliest nuclear events of HIV-1 infection and identifying new opportunities for therapeutic intervention. Throughout the figure, arrows indicate the direction of a process or of intracellular trafficking; a red cross (✗) denotes a step that is blocked or inhibited—in panel (**C**), innate immune sensing that fails to be triggered; question marks indicate steps that remain hypothetical and have not yet been demonstrated experimentally. Colour coding of the individual components (HIV-1 core, viral RNA, cDNA, host proteins/factors, CPSF6, SRRM2, integrase, LEDGF/p75, nucleosome and DNA methylation) is given in the key at the bottom of the figure.

The reconceptualised pathway can be summarised as a sequence of phase separation events (Figure 1). The selective barrier of the NPC central channel is itself a condensate, built from nucleoporins whose intrinsically disordered phenylalanine–glycine (FG) repeats undergo phase separation. Two independent studies showed that the HIV-1 capsid behaves like a nuclear transport receptor, partitioning into this FG phase by engaging specific hydrophobic pockets on the capsid lattice and crossing the pore much as a karyopherin would, even independently of classical nuclear transport receptors [22,23]. That the core fits at all is explained by the realisation, obtained once NPCs were studied in their native environment, that the central channel is substantially wider and more dilatable than once believed [24]. The capsid thus behaves as its own chaperone, its interior serving as a cargo container for the genome and viral enzymes.

**Figure 2:**
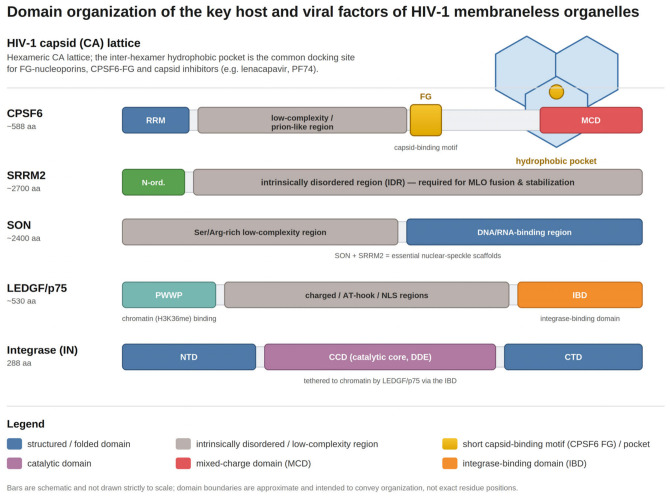
Domain organisation of the key host and viral factors of HIV-1 membraneless organelles. Schematic linear domain maps of the viral and host proteins whose interaction sites are described in the text. The HIV-1 capsid (CA) lattice presents a conserved inter-hexamer hydrophobic pocket that is engaged in turn by FG-nucleoporins, by the FG peptide of CPSF6 and by capsid inhibitors. CPSF6 combines an N-terminal RNA-recognition motif (RRM), a low-complexity/prion-like region, the short FG peptide that binds the capsid, and a C-terminal mixed-charge domain (MCD). The nuclear-speckle scaffolds SRRM2 and SON are dominated by long intrinsically disordered and low-complexity regions; the SRRM2 intrinsically disordered region (IDR) is required for fusion of HIV-1-MLOs with nuclear speckles and for their stabilisation. LEDGF/p75 bridges chromatin (via its PWWP domain) and integrase (via its integrase-binding domain, IBD). Integrase (IN) comprises an N-terminal domain (NTD), a catalytic core domain (CCD, DDE motif) and a C-terminal domain (CTD). Bars are schematic and not drawn strictly to scale, and domain boundaries are approximate.

Once in the nucleoplasm, the FG domain of CPSF6 binds the same inter-hexamer pockets, displacing the FG-nucleoporins and nucleating the HIV-1-MLO [25]. The recent genetic dissection of CPSF6 is instructive about the grammar of this assembly: the short FG peptide is both necessary and sufficient to bind the viral core and to induce puncta, and a CPSF6 variant lacking only the FG peptide fails to form HIV-1-MLOs and supports markedly less replication [20]. The domain organisation of these viral and host factors and the interaction sites discussed in this section are summarised schematically in Figure 2. The flanking low-complexity, prion-like regions tune this interaction rather than create it: their removal paradoxically increases capsid binding, implying that they modulate avidity [20,26]. The mixed-charge domain, by contrast, is dispensable for condensate formation in macrophage-like cells, a conclusion that differs from reports in tagged, HEK293-derived systems and underscores how cell context and protein tagging shape the readout [20,27]. These apparent contradictions are best read as consequences of methodology rather than of biology. Reports that assign a dispensable role to the mixed-charge domain rely largely on endogenous, untagged CPSF6 in primary or macrophage-like cells assessed by imaging, whereas those that place it at the centre of higher-order capsid binding and integration typically used over-expressed, fluorescently tagged CPSF6 in HEK293-derived lines and biochemical or reconstituted assays. Protein tagging can alter multivalency and phase behaviour, over-expression shifts the concentration regime in which condensates form, and the assays differ in what they quantify—puncta number and size by microscopy versus binding stoichiometry or integration frequency by biochemistry—so that thresholds are not directly comparable across studies. Reconciling these datasets will require standardised, endogenously tagged systems and matched quantification across cell types before the mixed-charge domain, and CPSF6 more broadly, can be assigned a single, context-independent function.

HIV-1-MLOs do not act in isolation; they fuse with nuclear speckles (NSs). Live imaging shows that CPSF6 condensates first form independently and then progressively coalesce with speckles, enlarging them as part of the hijacking process [20]. The intrinsically disordered region of the speckle scaffold SRRM2 (with SON, the two factors essential for speckle biogenesis [28]) is required for this fusion and for condensate stabilisation, whereas the mixed-charge cohesion domains implicated in speckle condensation are not needed for the viral structures [20,29]. The functional consequence is spatial: by docking at speckles, the virus completes nuclear reverse transcription within the MLO and then exports double-stranded DNA as a PIC primed for integration into speckle-associated domains (SPADs)—the LEDGF/p75-rich, gene-dense, transcriptionally active chromatin that favours high proviral output [8,14,30]. When the capsid–CPSF6 interaction is abolished, integration is redirected towards repressive lamina-associated domains, illustrating how the condensate dictates the genomic fate of the provirus [14,30]. The viral enzymes that act during these early steps—reverse transcriptase (RT) and integrase (IN)—enter the nucleus pre-formed within the incoming virion, so that de novo translation of viral proteins neither is required for nor occurs during these nuclear events; translation of the viral genome begins only later, after integration and proviral transcription. Integrase (IN) itself contributes to this phase-separated logic, forming condensates with its chromatin-tethering cofactor LEDGF/p75 (lens epithelium-derived growth factor/p75)—the IN–LEDGF/p75 complex—that modulate catalytic activity [31].

Two observations elevate these structures from in vitro curiosities to physiologically meaningful organelles. First, HIV-1-MLOs were imaged for the first time in vivo, in the tissues of infected humanised mice, in cells harbouring only incoming RNA as well as in productively infected cells [19]. Second, MLOs were shown to be the principal sites of nuclear reverse transcription for wild-type virus: pharmacological disassembly abolishes nuclear reverse transcription even when a reversible reverse-transcriptase inhibitor is withdrawn, a manoeuvre that normally restores the reaction inside intact MLOs [18,19]. The appearance of “empty” cores further supports the argument that uncoating is a regulated, nuclear, and possibly partial process, rather than the wholesale cytoplasmic shedding of the classical model [10,19].

### 2.1. Hijacking the Host: Molecular Piracy of Cellular Phase Separation Machinery (Figure 1A)

A striking feature of this biology is that the virus builds almost nothing de novo; it pirates pre-existing host condensate machinery and redirects it to viral ends. CPSF6 is, in uninfected cells, a paraspeckle-associated component of the cleavage factor I complex that governs alternative polyadenylation; on infection it is recruited away from paraspeckles and forced to phase-separate around the capsid lattice in speckle-proximal niches [14,16,17,32]. The scaffolds that build nuclear speckles, SON and SRRM2, are then conscripted to stabilise and enlarge the viral organelle: depleting SRRM2 or truncating its disordered C-terminus reduces HIV-induced puncta, so the virus co-opts the host’s own splicing factory as its workshop [20,28,29]. Upstream, the capsid exploits the FG-nucleoporin condensate of the pore as a permeability phase to dissolve into [22,23]; downstream, LEDGF/p75 tethers integrase to chromatin and itself supports IN–LEDGF condensation [30,31].

This strategy is strikingly economical. The virus encodes only minimal disordered determinants, the hydrophobic pockets distributed across the CA hexamer lattice and the short FG peptide of CPSF6, which act as multivalent nucleation seeds that lower the threshold for host factors to phase-separate where and when the virus requires [18,20,33]. HIV-1 does not so much invent an organelle as bias the host’s intrinsic phase separation equilibria. This is a specific instance of a general viral tactic: many viruses hijack or remodel host condensates (stress granules, paraspeckles, speckles, promyelocytic leukaemia nuclear bodies) to concentrate their machinery and neutralise antiviral nodes [11,13]. Recognising HIV-1-MLOs as repurposed host condensates rather than purely viral structures reframes the therapeutic problem: the targets are largely cellular, which raises both opportunities (host-directed, mutation-resistant intervention) and hazards (on-target toxicity to essential nuclear compartments).

### 2.2. Condensates as Catalytic Crucibles: Reverse Transcription and Productive Replication (Figure 1B)

Biomolecular condensates accelerate biochemistry by concentrating reactants and selectively excluding others, creating local microenvironments in which reaction rates and specificities differ from those in the bulk nucleoplasm [33]. HIV-1-MLOs exemplify this principle in the service of the virus. They co-concentrate the incoming genome inside viral cores and presumably the deoxynucleotide substrates, converting a dilute and inefficient nuclear reaction into a clustered, productive one [14,15,18]. Because reverse transcriptase and integrase are delivered pre-assembled within the capsid, the reaction demands no local protein synthesis; what the compartment must instead supply is the substrate pool. How deoxynucleoside triphosphates (dNTPs) are concentrated within the MLO is not yet established, but their nuclear availability is set by SAMHD1-dependent catabolism and may be governed by the same partitioning properties that admit small molecules into condensates, so that the niche could act on substrates and inhibitors alike. Defining where reverse transcription physically occurs—within the capsid interior, at its surface, or in the surrounding condensate—and how dNTPs reach that site are open questions that follow directly from this model. The functional proof is direct: dismantling the condensate abolishes nuclear reverse transcription even after a reversible inhibitor is withdrawn, whereas in an intact niche the reaction resumes [18,19]; the FG peptide that nucleates the condensate also facilitates replication, so that CPSF6 variants lacking it both fail to form puncta and support substantially less infectivity and progeny production [20].

Replication is favoured a second time at the level of integration. By docking the PIC at nuclear speckles and channelling integration into LEDGF/p75-rich SPADs, the condensate places the provirus in gene-dense, transcriptionally permissive chromatin that maximises viral output, whereas loss of the capsid–CPSF6 interaction diverts integration to repressive compartments and lowers expression [14,30]. The arrangement is dual-use: the same compartment that accelerates reverse transcription and optimises integration also, as the next section argues, shields the most immunostimulatory product of that reaction, so that replication efficiency and stealth are delivered by a single HIV-1 MLO.

### 2.3. Condensates as Innate Immune Shields: A Layered Evasion Strategy (Figure 1C)

HIV-1 is a notoriously poor activator of innate immunity during the early phase of infection [34], and this quiescence is the result not of a single trick but of a stratified programme in which the nuclear condensate is the last layer. In the cytoplasm, the host deploys several barriers that the virus must first survive. The cyclic GMP–AMP synthase (cGAS) is the principal sensor of retroviral cDNA [35], but the exonuclease TREX1 degrades cytosolic reverse-transcription by-products before they accumulate to immunostimulatory levels, blunting cGAS activation [36]; SAMHD1 depletes the dNTP pool in myeloid and resting cells, throttling reverse transcription itself [37]; the interferon-induced MX2/MxB restricts nuclear import and uncoating by engaging the capsid [38]; and TRIM5 recognises the capsid lattice and can function as a pattern-recognition receptor that triggers innate signalling [39]. In lymphoid CD4 T cells abortively infected with HIV, the DNA sensor IFI16 detects incomplete reverse transcripts and drives inflammatory, pyroptotic death [40]. Pandemic HIV-1 has been selected, in part, for its capacity to evade cGAS and TRIM5 [41].

The nucleus poses a distinct problem. Although the host factor NONO (non-POU-domain-containing octamer-binding protein, a paraspeckle DNA- and RNA-binding factor) can detect the nuclear capsid and promote cGAS activation [42], nuclear cGAS is normally held catalytically inert by tight, salt-resistant tethering to chromatin and senses only non-chromatinised DNA [43,44,45]. HIV-1 generates exactly such a ligand—double-stranded viral DNA synthesised in the nucleus before it can be chromatinised—and so should, in principle, be visible. The resolution is that the condensate is the final shield: when HIV-1-MLOs are dismantled, with the capsid-binding compound PF74 or in CPSF6-knockout cells, the cGAS–STING axis is activated and interferon-stimulated genes are induced, whereas intact MLOs keep the genome sequestered and the sensor silent [18,19]. Published in vivo data indicate that it is the double-stranded DNA product, rather than incoming single-stranded RNA or RNA/DNA hybrids, that would trigger cGAS, which couples the timing of reverse transcription and the integrity of the condensate tightly to immune escape [19]. The layers thus operate as a relay: cytoplasmic restriction is met by completing reverse transcription in the nucleus, and nuclear sensing is met by concealing the product within a condensate until it can be integrated and chromatinised.

### 2.4. Convergent Evolution: Viral Condensates as Immune Sanctuaries Across Families (Figure 1D)

The HIV-1 strategy is a specialised instance of a broadly conserved theme. The clearest contemporary parallel is SARS-CoV-2, whose nucleocapsid (N) protein undergoes RNA-driven phase separation that promotes genome packaging and replication [46,47] and, by the same condensation, mediates immune evasion: N partitions into and disrupts G3BP1-containing assemblies, impairing the cGAS–G3BP1 cofactor complex and blunting cytosolic DNA sensing of mitochondrial DNA released during infection [48], and it suppresses the prion-like condensation of MAVS required for RNA-sensing signal transduction [49]. The host can retaliate in the same currency: cellular nucleic-acid-binding protein restricts SARS-CoV-2 partly by dissolving these N–RNA condensates and restoring interferon [50]. The structural details differ, with SARS-CoV-2 acting in the cytoplasm on RNA-sensing and cGAS-cofactor hubs while HIV-1 acts in the nucleus on the cGAS–DNA reaction, but the logic is identical: phase separation is used to exclude or sequester a sensor away from its ligand.

The negative-strand RNA viruses provide the historical template. Rabies virus builds Negri bodies, liquid viral factories that also blunt interferon induction; vesicular stomatitis virus assembles replication compartments by phase transition; and respiratory syncytial virus and Ebola virus form inclusion bodies that, respectively, sequester the dsRNA sensor MDA5 with its adaptor MAVS and re-route the transcription factor IRF3 away from its duties [51,52,53,54,55]. Across these systems two complementary tactics recur: sequestration-in, whereby a sensor is dragged into the viral condensate and inactivated, and exclusion-out, whereby the immunostimulatory nucleic acid is hidden inside the condensate away from a sensor that remains active elsewhere. HIV-1-MLOs are the nuclear, exclusion-out exemplar. A further shared feature is conditionality: stress can reverse the quiescence of condensate-resident genomes, as shown most vividly for mumps virus, whose dormant genomes are reactivated by a stress-induced condensate switch [56,57]. If the HIV-1 genome can persist in a stable MLO and be reawakened by environmental change, the condensate is not only a shield but also a switch, with potential implications for the viral genome fate.

### 2.5. From Nuclear Sanctuaries to Viral Reservoirs and the Establishment of Latency (Figure 1E)

The link between a transient nuclear condensate and the durable reservoir that defies cure is being closed experimentally. A new imaging platform engineers replication-competent HIV-1 carrying the bacterial-derived ANCHOR system (a ParB–parS DNA-labelling system adapted from bacterial chromosome-segregation machinery, in which a fluorescently tagged ParB protein binds and oligomerises on an integrated parS sequence), in which this fluorescent protein decorates the integrated tag and renders individual double-stranded viral DNA (vDNA) molecules visible as nuclear puncta; because the tag is read out only from double-stranded intranuclear DNA, the signal reports genuine reverse-transcription products rather than abortive particles [21]. Combined with single-molecule RNA FISH, this allows for transcribing and silent genomes to be distinguished within single CD4^+^ T cells across organs during acute infection. The distribution is tissue-specific: actively transcribing viral DNA predominates in the spleen (around 69% of vDNA-positive cells), is intermediate in the bone marrow (around 56%), and is lowest in the lymph nodes (around 14%), with silent forms correspondingly enriched in lymph nodes and marrow [21]. Transcriptionally quiescent, potentially long-lived genomes are therefore seeded early and preferentially in specific niches, precisely the behaviour expected of a nascent reservoir.

Two forms of latency must be distinguished, and the condensate is relevant to both. Pre-integration latency reflects unintegrated viral DNA, which is loaded with core and linker histones and transcriptionally silenced yet capable of transient expression [58]; the stable, weeks-long persistence of viral cargo within HIV-1-MLOs makes the condensate a plausible pre-integration reservoir, distinct from but upstream of the integrated pool [18]. Post-integration latency, the principal barrier to cure, is shaped at the moment of integration: by routing the provirus into active SPADs or, when disrupted, into repressive chromatin, the condensate could be a decision point that sets the transcriptional fate and reactivation potential of the provirus [14,30]. A decisive functional experiment showed that pharmacological dismantling of CPSF6 puncta prevents the restoration of nuclear reverse transcription after withdrawal of a reversible inhibitor; only when the niche is intact does the trapped genome resume reverse transcription [14,20].

## 3. Clinical Implications

### 3.1. Plasma HIV-1 RNA Dynamics, Residual Viraemia, and Rebound

If a stable nuclear condensate can trap a reverse-transcription-competent genome and release it when conditions permit, the condensate becomes directly relevant to the quantity clinicians monitor most closely: plasma HIV-1 RNA. The in vitro analogue is explicit: trapping incoming genomes with a reversible reverse-transcriptase inhibitor and then removing the drug allows reverse transcription to resume inside the niche, but only if the niche persists [14,20]. Extrapolated cautiously to people on suppressive therapy, this offers a mechanistic candidate for part of the residual viraemia and the low-level “blips” that punctuate otherwise undetectable viral loads, and for the kinetics of rebound after treatment interruption. The hypothesis to be tested is that the pre-integration, condensate-resident pool contributes to the earliest phase of rebound, before expansion of the integrated reservoir dominates; agents that dissolve or harden the niche might then alter rebound timing independently of their effect on the integrated provirus.

### 3.2. Antiretroviral Drug Penetration: Anatomical and Molecular Sanctuaries

A sanctuary that excludes immune sensors may also exclude drugs, and here two nested levels deserve attention. The anatomical level is established: antiretroviral concentrations are substantially lower in lymphoid tissues than in blood, by as much as an order of magnitude or more for several first-line agents, and these lower concentrations correlate with persistent virus replication in lymph nodes despite undetectable plasma viraemia [59]. Lymph nodes and gut-associated lymphoid tissue thus act as pharmacologic sanctuaries in which incomplete drug penetration permits a smouldering, ongoing cycle of infection [60,61]. The in vivo imaging discussed above adds anatomical resolution to this picture, showing that the most transcriptionally active genomes reside in the spleen while quiescent forms accumulate in lymph nodes and marrow [21].

The molecular level is, by contrast, hypothetical but increasingly plausible. Biomolecular condensates selectively partition or exclude small molecules according to physicochemical properties that are independent of target engagement; in transcriptional condensates, for example, cisplatin is concentrated several hundredfold, and condensate partitioning is now recognised as an axis of drug pharmacodynamics and an emerging principle of drug discovery [62,63]. By extension, HIV-1-MLOs, being phase-separated nuclear compartments with a distinct chemical environment, may differentially admit antiretrovirals, concentrating some agents (plausibly the capsid inhibitors that must reach the CA pocket within the condensate [16,17]) while excluding others, so that the effective intra-condensate exposure of a drug diverges from its bulk intracellular concentration. If this proves true, the molecular sanctuary would compound the anatomical one: the very reverse-transcription reaction that the condensate accelerates could be sheltered from a nucleoside or non-nucleoside inhibitor that is formally present in the cell but partitioned away from the niche. The proposition is directly testable, by measuring the condensate partition coefficients of antiretrovirals in reconstituted CPSF6/capsid condensates and by intranuclear imaging, and it carries a design corollary: antiretrovirals, and capsid inhibitors in particular, might be optimised not only for target affinity but for favourable partitioning into the viral condensate. We emphasise that no direct measurement of antiretroviral partitioning into HIV-1-MLOs yet exists; this is offered as a research hypothesis.

### 3.3. A Speculative Link to Coreceptor (R5 → X4) Evolution

In the spirit of a Perspective we offer the following as a deliberately speculative assessment. The switch in coreceptor usage from CCR5 (R5) to CXCR4 (X4), determined chiefly by sequence changes in the V3 loop of gp120, emerges in roughly half of subtype-B infections during the course of disease and is robustly associated with accelerated CD4^+^ T-cell decline and progression to AIDS [64,65]. Two threads connect this phenomenon to the condensate biology. First, immune activation precedes and independently predicts the X4 switch [65], and the persistence of cryptically replicating or low-level-expressing genomes in sanctuary niches (the spleen and marrow being the most transcriptionally active in vivo [21], the lymph nodes the least drug-exposed [59]) could sustain the smouldering antigenic and inflammatory milieu that the activation correlate reflects, pointing to a feed-forward loop in which sanctuary-enabled persistence fuels the activation that selects for X4. Second, because the condensate dictates integration site choice and the transcriptional fate of the provirus [14,30], and because reactivation from these niches is conditional [56,57], the MLO may bias which archived variants, R5 or X4, are preferentially reawakened and re-seeded. No current data directly connect HIV-1-MLOs to coreceptor switching; the value of the hypothesis is that it is testable, for instance by coupling longitudinal tropism genotyping to organ-resolved imaging of transcribing versus silent genomes [21].

### 3.4. Diagnostic Perspectives

The most immediate translational yield is diagnostic. The ability to visualise, and to classify as transcribing or silent, individual viral DNA molecules across tissues [21] points towards reservoir profiling that captures not merely the size but the transcriptional state and anatomical distribution of persistent virus. Such single-cell, spatially resolved read-outs would complement the molecular assays that increasingly anchor reservoir research: intact proviral DNA quantification and the phenotypic and epigenetic signatures that distinguish reservoir cells [66,67]. Given the finding that silent genomes are enriched in lymph nodes and marrow while active genomes dominate the spleen [21], together with the lower drug exposure of lymphoid tissue [59], we caution that blood-based sampling may systematically under-represent the most quiescent and least-treated compartments and argue for tissue-aware interpretation of any reservoir biomarker. In principle, the abundance of nuclear condensates, or of their non-chromatinised DNA cargo, could itself serve as a marker of functional, reactivation-prone infection [18]. These remain research-grade approaches, developed in humanised mice rather than patients, and their translation will require validation against clinical endpoints and accessible specimens.

## 4. Therapeutic and Vaccine Perspectives

The discovery that a druggable viral structure organises reverse transcription, integration, and immune concealment reframes the capsid as a multi-purpose target. Long considered undruggable, the capsid yielded to lenacapavir, a long-acting inhibitor that interferes with multiple steps of the life cycle [68]; lenacapavir is approved for multidrug-resistant infection [69] and, since 2025, as twice-yearly subcutaneous pre-exposure prophylaxis on the strength of the PURPOSE trials [70]. Mechanistically, capsid-binding compounds occupy the pocket that CPSF6 uses to nucleate HIV-1-MLOs; PF74 and related ligands compete with CPSF6 and disassemble pre-formed condensates [16,17]. This convergence suggests a repurposing logic: capsid inhibitors are not only entry-to-integration blockers but also condensate disruptors, and disrupting the MLO unmasks viral DNA to cGAS–STING [18,19]. A “block-and-expose” immune-sensitising strategy would deliberately dismantle the nuclear sanctuary to convert a silent, shielded genome into an immunostimulatory one, recruiting innate immunity to flag infected cells, provided the kinetics, tissue penetration, and inflammatory balance can be controlled.

The condensate also offers handles for the opposite block-and-lock philosophy, which seeks durable, drug-independent transcriptional silencing of the provirus rather than its elimination [71,72]. Because the MLO routes integration towards transcriptionally active speckle-associated chromatin, agents that redirect integration—most notably LEDGINs, which disrupt the integrase–LEDGF/p75 interaction and shift residual proviruses into repressive, reactivation-refractory chromatin [73]—are conceptually complementary to condensate modulation, and CPSF6-dependent integration-site selection is now explicitly discussed in this context [74].

Finally, there are implications for vaccination. A virus that has evolved to phase-separate its genome away from nucleic-acid sensors begins each infection with a muted innate response [34,41], and a weak innate response is a poor adjuvant for adaptive immunity. If condensate-mediated concealment contributes to the blunted early interferon signature of HIV-1, then lifting that concealment during acute infection, as part of a therapeutic-vaccine prime, or as an adjunct to latency reversal, could enhance antigen availability and innate priming. The cross-viral perspective reinforces the point: the immune-evasiveness of several viruses tracks with their capacity to suppress interferon through condensates [48,49], so condensate disruption could be a global immunopotentiation strategy rather than an HIV-specific trick. These ideas are hypotheses, not recommendations, and will require careful evaluation of the balance between productive immune sensing and pathological inflammation.

## 5. Concluding Remarks and Future Directions

The past five years have rewritten what is known about the opening chapter of the HIV-1 life cycle, moving the decisive events from the cytoplasm to the nucleus and from diffuse biochemistry to organised, phase-separated compartments. Viewed across the five axes developed here, HIV-1-MLOs emerge as pirated host condensates (hijacking) that act as catalytic crucibles for reverse transcription and high-output integration (replication), as the final shield of a layered programme that defeats cytoplasmic and nuclear sensors alike (immune evasion), as the seed of the pre- and post-integration reservoir (latency), and, in a dimension that still deserves attention, as a potential pharmacological barrier nested within the anatomical lymphoid sanctuaries (drug access). That a betacoronavirus reached a functionally analogous solution by independent means suggests that condensate-mediated concealment is a recurrent, perhaps inevitable, evolutionary answer to nucleic-acid surveillance.

Three priorities follow. First, the field needs patient-relevant tools: the in vivo imaging and condensate read-outs developed in humanised mice must be adapted to human specimens and integrated with intact-proviral-DNA and single-cell reservoir assays, to test whether nuclear sanctuaries shape residual viraemia, rebound, drug response and, more speculatively, coreceptor evolution. Second, the pharmacology of the condensate must be measured directly: do antiretrovirals partition into or out of HIV-1-MLOs, and can capsid inhibitors and condensate-disrupting agents be designed accordingly? Third, the therapeutic logic of the capsid must be pursued in both directions, as a block-and-expose immune-sensitiser and, with integration-site modulators, as part of a block-and-lock silencing programme, alongside quantitative definition of the immunological consequences of dismantling viral condensates. The virus has, for four decades, hidden in plain sight within the nucleus; the tools to visualise it, to reach it with drugs, and to decide whether to expose it or to lock it away are now in hand.

## Data Availability

No new data were created or analysed in this study. Data sharing is not applicable to this article.
